# Clinical application of color Doppler ultrasound for assessing hemodynamic changes in the children with moyamoya disease undergoing combined revascularization surgery

**DOI:** 10.3389/fped.2025.1526900

**Published:** 2025-04-28

**Authors:** Chenyun Zhou, Hang Ji, Hongxia Fan, Yue Li, Lina Han, Anqi Xiao, Xiaoxia Zhu, Haogeng Sun, Zhizhi Tan, Ying He, Yi Liu

**Affiliations:** ^1^ Department of Medical Ultrasound, West China Hospital, Sichuan University, Chengdu, Sichuan, China; ^2^ Department of Neurosurgery, West China Hospital, Sichuan University, Chengdu, Sichuan, China

**Keywords:** pediatric, moyamoya disease, color Doppler ultrasound, superficial temporal artery, carotid artery, hemodynamics

## Abstract

**Objective:**

To investigate the advantages of color Doppler ultrasound (CDUS) in detecting hemodynamic alterations in children with moyamoya disease (MMD) following combined revascularization surgery.

**Methods:**

The common carotid artery (CCA), internal carotid artery (ICA), external carotid artery (ECA), and superficial temporal artery (STA) were measured by CDUS. Hemodynamic parameters including arterial diameter, peak systolic velocity (PSV), resistance index (RI), and blood flow volume (FV) were collected at three time points: pre-operation (T1), one week after operation (T2), and three months after operation (T3). Twelve children without intracranial arterial disease were recruited as the control group. Matsushima classification-based on digital subtraction angiography (DSA) was applied at T2.

**Results:**

Among the 12 children with MMD, 11 patients with bilateral arterial stenosis and 1 patient with unilateral being affected. Compared to the 24 control hemispheres, the diameter of the ICA was significantly smaller in the 23 MMD hemispheres (*p* < 0.001) with an increased PSV of CCA and ECA, and a decrease FV of carotid arteries (*p* < 0.05). In MMD group, CDUS revealed increased diameter and FV, decreased RI of STA at the operative side at T2. The PSV and FV of ECA at the operative side increased from T1 to T3 (*p* < 0.05). Six cases were allocated to satisfactory compensation group (S Group, Matsushima classification grade A and B) and six cases to dissatisfactory compensation group (DS Group, Matsushima classification grade C). The increase in FV of STA on the operative side was higher in S Group at T2 than DS Group (Spearman rho = −0.693, *p* = 0.039).

**Conclusion:**

As a noninvasive imaging modality, carotid and superficial temporal arteries ultrasound may serve as a valuable adjunct to invasive imaging techniques for children with MMD.

## Introduction

Moyamoya disease (MMD) is a leading cause of pediatric stroke, characterized by a non-inflammatory, non-atherosclerotic chronic stenotic and occlusive vasculopathy. The disease predominantly affects the terminal portion of the internal carotid artery and the origin of the middle cerebral artery (MCA) and anterior cerebral artery, while rarely involving the posterior cerebral artery (1). As a result, the gradual compensation of patients' cerebral blood supply is facilitated by the external carotid and vertebrobasilar artery systems. During the early stages of the compensatory transition, intracranial ischemia may intensify, resulting in the formation of neovascularizations at the skull base. These formations present a radiographic appearance reminiscent of “smoke”, which gives rise to the term “Moyamoya”, derived from Japanese words meaning “hazy” or “puff of smoke”. Concurrently, collateral branches from the external carotid and vertebral arteries dilate progressively, forming a sophisticated network of collateral pathways to offset the ischemic impact (2, 3).

Diagnosis and assessment of MMD commonly rely on imaging modalities such as computed tomography angiography, computed tomography perfusion, and digital subtraction angiography (DSA) (2, 3), which are invasive and involve radiation exposure. The repeated use of these diagnostic tools to children presents greater challenges and risks compared to adults. Although magnetic resonance imaging and magnetic resonance angiography are generally regarded as safe and effective (2, 4), their extended duration often requires pediatric patients to remain still or undergo sedation, limiting their clinical feasibility. Given these limitations, there is a pressing need for noninvasive, rapid imaging modalities suitable for children with MMD.

Recent studies (5–9) have shown that color Doppler ultrasound (CDUS) of the carotid artery and superficial temporal artery (STA) can predict neovascularization and postoperative outcomes in MMD population. However, in children with MMD the predictive value and feasibility of CDUS was seldom discussed fully. This study aimed to assess whether various ultrasound parameters, measured after revascularization surgery in children with MMD, can faithfully reflect changes in blood flow dynamics. Additionally, we sought to identify which specific parameters best correlate with improved intracranial blood supply post-surgery.

## Materials and methods

### Patients and management

This study included children diagnosed with MMD by DSA at West China Hospital, Sichuan University, between January 2022 and June 2024. The inclusion criteria were set as: (1) age ≤ 16 years, (2) preoperative diagnosis confirmed by DSA imaging, (3) unilateral extracranial-intracranial arterial bypass surgery without treatment on the contralateral side, (4) CDUS assessments at baseline and 7 days postoperatively or 3 months postoperatively, and (5) completion of DSA 7 days postoperatively. The exclusion criteria included: (1) refusal of revascularization surgery by patients or guardians, (2) revascularization surgery performed on the contralateral side before or within 3 months postoperatively, (3) incomplete preoperative or postoperative ultrasound assessments, and (4) failure to complete postoperative DSA. A control group included children without hematological, cardiovascular, neurological, or immune disorders, presenting for unrelated conditions. A written informed consent was obtained from all participants and/or their guardians. All procedures performed in this study were in accordance with the ethical standards of the Ethical Committee of West China Hospital.

### Brief description of surgical procedure

A combined revascularization procedure, incorporating both a direct STA to MCA bypass and an indirect encephalo-duro-arterio-synangiosis bypass, was performed on all patients. The frontal and parietal branches of the STA were dissected from the scalp flap and employed as donor arteries. The anastomosis between a branch of the STA and the MCA was executed in an end-to-side manner using a micromanipulation microscope. The selection of the recipient artery was determined microscopically, based on criteria such as regions of reduced cerebral blood flow, diameter, and donor accessibility. Following successful anastomosis, intraoperative indocyanine green fluorescence angiography was performed to assess graft patency. Upon confirmation of anastomotic patency, the dura mater was inverted and adhered to the brain surface, and the temporalis muscle was sutured to the dura at the periphery of the bone window. Subsequently, the bone flap was trimmed, repositioned, and secured. All combined revascularization procedures were conducted by a single experienced surgeon.

### Ultrasound assessment

Ultrasonographic examination was performed using EPIQ7 US system (hilips Healthcare, Bothell, WA) with an eL18-4 linear array transducer and a L12–3 linear array transducer, detecting STA and carotid arteries respectively. The arteries were measured at selected locations: the distal segment of the CCA about 1–2 cm below the bifurcation, the proximal segment of the ICA and the ECA about 1–2 cm above the bifurcation. The STA was measured at the temporal region, above and in front of the ears. Selected a segment with a relatively straight arterial course for measurement, avoiding the segment with carotid sinus and arterial branches.

Measurements included arterial diameter, peak systolic velocity (PSV), resistance index (RI), and blood flow volume (FV). Arterial diameters were measured as accurately as possible at the above location in grayscale mode. When the STA was too thin or too deep, Micro-flow imaging technology was used to better outline the blood flow morphology. Measured the width of the blood flow bundle by this technique as the arterial diameter. Then selected the Doppler mode, placed the sampling volume at the center of the arterial diameter and covered the entire inner diameter of the artery as much as possible. Accurately collected Doppler spectrum, measured PSV and end diastolic velocity, and marked the arterial diameter (using the data measured in grayscale mode). The machine would automatically calculate the RI and arterial FV according to the following formula.

RI:(PSV-EDV)/PSV

FV:D2×π/4×TAMV×60(ml/min)

(EDV: end diastolic velocity, D: Arterial diameter, TAMV: Time averaged mean flow velocity)

CDUS assessment of the carotid and temporal arteries were performed at three time points: pre-operation (T1), one week after operation (T2), and three months after operation (T3). Control group measurements were taken at comparable locations.

All our examinations were conducted by two experienced vascular ultrasound experts who had been engaged in vascular ultrasound for over 10 years, and had comparable levels of experience in conducting preoperative and postoperative ultrasound examinations for patients with MMD and Moyamoya arteriopathy. When measuring, we tried our best to ensure that the children were in a quiet condition. All included children were able to cooperate in completing the examination by diverting attention or rewarding candy, toys, etc., and no child used sedatives during the examination. During the examination, the ultrasound probes were placed gently at the examination site to avoid excessive pressure that might cause resistance in the patient and measurement errors caused by corresponding pressure. Warm medical coupling gel was used to avoid arterial constriction caused by cold, which may affect the measurement results, especially for superficial temporal arteries. When significant abnormalities were detected in the measurement, immediately repeated the measurement three times and selected the median as the measurement data.

### Matsushima grading

Postoperative DSA was conducted on day 7, and Matsushima grading was performed according to the DSA imaging as Grade A, B and C. The perfusion area within the graft in the MCA supply region was evaluated as follows: Grade A (≥2/3 MCA perfusion area), Grade B (1/3–2/3), and Grade C (<1/3). Grades A and B were considered satisfactory compensation (S group), and Grade C was designated as dissatisfactory compensation (DS group). Matsushima grading was independently performed by two neurovascular surgeons, with a third senior surgeon resolving any controversy.

### Statistical analysis

Data analysis was conducted using SPSS (v22). Data that conformed to a normal distribution was described statistically using mean ± standard deviation, with non-normally distributed data reported as medians and interquartile ranges or medians and ranges. Paired *T*-tests were used to compare preoperative blood flow parameters of the MMD group with those of the control group. Wilcoxon rank sum test was used to compare ultrasound parameters between different time points and between the left and right sides of the MMD group. Mann–Whitney *U*-test was used to compare the increase of FV in STA between the S group and the DS group. Spearman correlation was used for correlation analysis between the percentage increase of FV of the STA with Matsushima grading. A *p*-value of <0.05 was considered statistically significant.

## Results

### Clinical characteristics

Of the 20 children with MMD, 12 met the inclusion criteria (7 males, 5 females). Four patients had a history of cerebral infarction, and eight demonstrated hypoperfusion on imaging (Table 1). The surgical procedure was executed with precision, and the patient remained free from postoperative vascular complications. Nine and ten patients completed CDUS follow-up at T2 and T3, respectively. All patients' blood pressure was well controlled with medication and the difference before and after surgery was within 20 mmHg to ensure comparability.

**Table 1:** Fundamental clinical characteristics of the patient cohort (*n* = 12).

Variables	Values
Affected Side (Number of Cases, %)	
Unilateral	1 (8.3%)
Bilateral	11 (91.7%)
Primary Symptoms at Onset	
Limb Weakness	5 (41.7%)
Headache, Nausea	4 (33.3%)
Dizziness, Speech Impairment	1 (8.3%)
Syncope, Intermittent Convulsions	1 (8.3%)
No obvious symptoms	1 (8.3%)
mRS Score	
0–1 Points (%)	10（83.3%）
≥2 Points (%)	2 (16.7%)
Clinical Type	
Ischemic Type	12 (100%)
Hemorrhagic Type	0
Suzuki Grading	
Grade III (%)	9 (75.0%)
Grade IV (%)	3 (25.0%)
Surgical Side (Number of Cases, %)	
Left Side	5 (41.7%)
Right Side	7 (58.3%)

The control group consisted of 12 children (6 males, 6 females) with various unrelated conditions (e.g., post-surgical follow-up, hydronephrosis, thyroid nodules). All participants had normal blood tests and fully cooperated during ultrasound assessments (Table 2).

**Table 2:** Comparison of basic clinical characteristics between the MMD group and the control group.

Clinical characteristics	MMD group	Control group	*p*
Sex, Number of Cases (%)			0.698
Male	7 (58.3%)	6	
Female	5 (41.7%)	6	
Age, years, Median (Min, Max)	8 (3,16)	7.5 (3,15)	0.850
Body Mass Index, kg/m^2, Median (Min, Max)	17.2 (12.3,31.7)	15.6 (13.7,20.8)	0.134

### Comparison of CDUS parameters between MMD hemispheres and control hemispheres

Compared 23 affected cerebral hemispheres in the MMD group to 24 normal cerebral hemispheres in the control group, reduced ICA diameter (*p* < 0.001), elevated PSV in the CCA and ECA (*p* < 0.001), increased RI in the CCA (*p* < 0.01), and decreased FV in all carotid arteries (*p* < 0.05) was shown on preoperative CDUS in the MMD hemispheres. The FV of CCA, ICA, and ECA decreased by 25.8%, 50.6%, and 46.4% respectively with PSV of the STA elevated by 61.4% compared to controls (Table 3).

**Table 3:** Comparison of blood flow parameters between MMD hemispheres (*n* = 23) and control hemispheres (*n* = 24) (mean ± standard deviation).

CDUS parameters	Control Hemispheres	MMD Hemispheres	*p*
CCA			
Diameter (mm)	5.4 ± 0.4	4.9 ± 1.1	0.5
PSV (cm/s)	132.6 ± 23.5	155.8 ± 28.8	0.004*
RI	0.72 ± 0.05	0.77 ± 0.05	**0**.003*
FV (ml/min)	479.8 ± 87.1	356.2 ± 118.9	**0**.000*
ICA			
Diameter (mm)	4.4 ± 0.4	3.5 ± 0.6	**0**.000*
PSV (cm/s)	120.8 ± 36.1	109.5 ± 24.6	0.215
RI	0.65 ± 0.05	0.65 ± 0.05	0.825
FV (ml/min)	314.7 ± 72.4	155.4 ± 67.8	**0**.000*
ECA			
Diameter (mm)	3.5 ± 0.5	3.5 ± 0.6	0.891
PSV (cm/s)	92.4 ± 19.7	122.2 ± 23.7	**0**.000*
RI	0.83 ± 0.08	0.83 ± 0.10	0.994
FV (ml/min)	74.1 ± 105.6	39.7 ± 41.6	**0**.011*
STA			
Diameter (mm)	1.4 ± 0.2	1.3 ± 0.4	0.207
PSV (cm/s)	46.1 ± 13.7	74.6 ± 18.2	**0**.000*
RI	0.85 ± 0.08	0.85 ± 0.05	0.954
FV (ml/min)	8.4 ± 3.9	11.7 ± 8.9	0.115

CCA, common carotid artery; ICA, internal carotid artery; ECA, external carotid artery; STA, superficial temporal artery; PSV, peak systolic velocity; RI, resistance index; FV, arterial blood flow volume. *Represents *p* < 0.05, values of statistical significance are highlighted in bold.

### Comparison of CDUS parameters at three intervals in the MMD group

#### STA (the blood flow of bilateral STA increased at a time-dependent manner)

**Figure 1:**
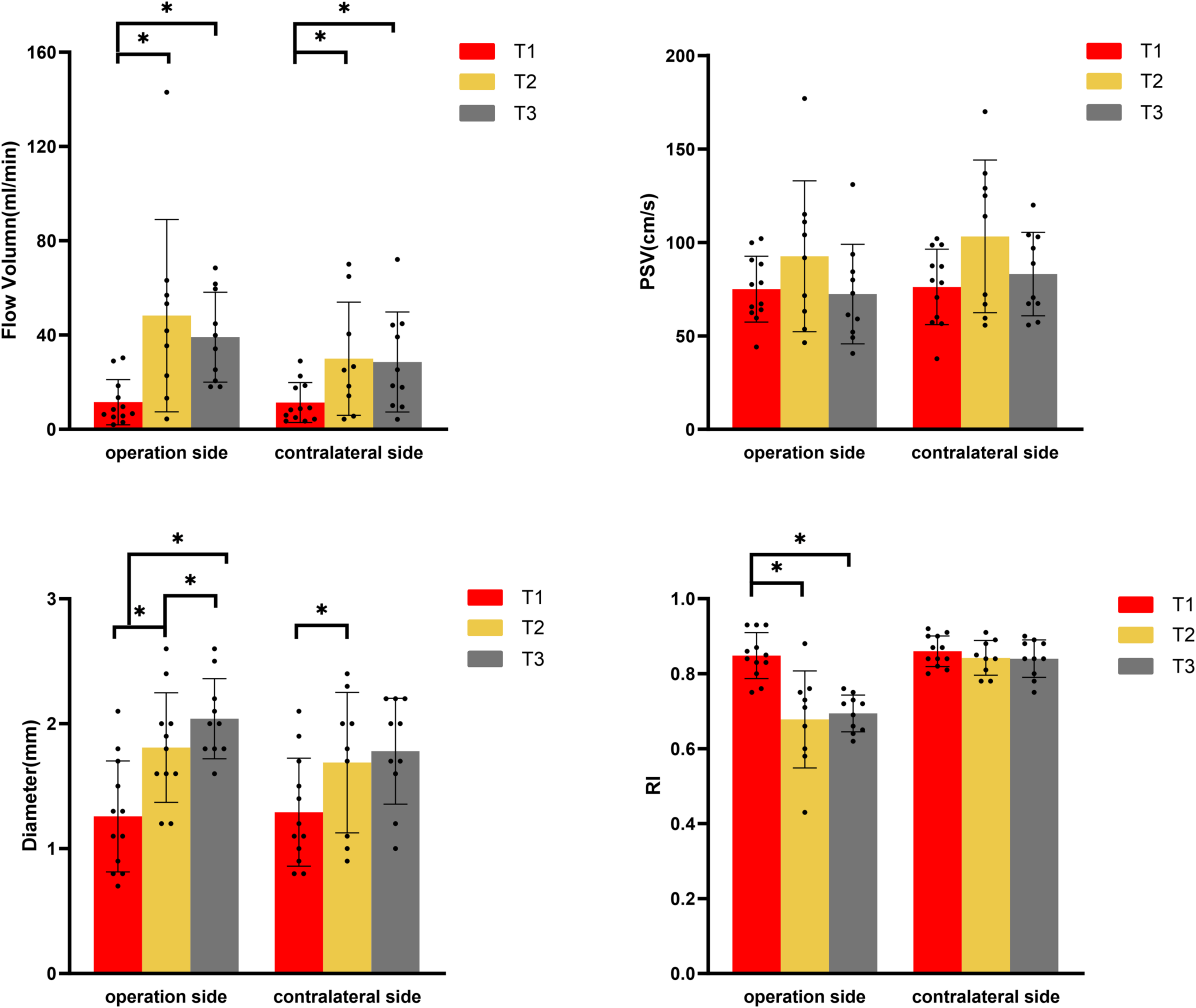
Changes of blood flow parameters of superficial temporal artery in moyamoya group at different time points by color Doppler ultrasound. (PSV, peak systolic velocity; RI, resistance index; T1, pre-operation; T2, one week after operation; T3, three months after operation). *Represents *p* < 0.05.

At T1, no statistically significant differences were observed in the ultrasound parameters between the bilateral STA. At T2, both DSA and CDUS confirmed that all graft arteries within the MMD group remained patent. The CDUS analysis revealed a significant increase in the diameter of the bilateral STA from T1 to T2, with *p*-values of 0.008 for the ipsilateral side and 0.027 for the contralateral side. Additionally, the FV also demonstrated a significant increase, with *p*-values of 0.011 for the ipsilateral side and 0.008 for the contralateral side. At the operative side, the mean increase in diameter was 0.6 mm (46.2%), and the mean increase in FV was 36.7 ml/min (319%). Intriguingly, the mean increase in diameter was 0.5 mm (39%), and the mean increase in FV was 18.8 ml/min (165%) at the contralateral side. The RI of the STA on the graft side exhibited an average decrease of 0.16 (19%), whereas no significant decrease was observed on the contralateral side. Although there was a trend towards increased PSV in the bilateral STA, this difference did not reach statistical significance (Figure 1). Upon comparing the graft side to the contralateral side at time point T1, no statistically significant differences were observed in any of the blood flow parameters of STA. However, at T2, a statistically significant difference was identified exclusively in the RI of STA. The RI of STA on the operation side was significantly lower than that on the contralateral side (Figure 1 and Table 4).

**Table 4:** Comparison of ultrasound parameters in bilateral superficial temporal arteries of the disease group. (medians, interquartile ranges).

Ultrasound parameters	Operative Side	Contralateral Side	*p*
T1			
Diameter (mm)	1.2 (0.8, 1.7)	1.2 (0.9, 1.7)	0.459
PSV (cm/s)	72.3 (62.8, 90.1)	79.1 (57.9, 95.8)	0.875
RI	0.85 (0.81, 0.92)	0.86 (0.83, 0.90)	0.478
FV (ml/min)	7.6 (5.3, 17.3)	8.6 (4.5, 18.4)	0.755
T2			
Diameter (mm)	1.8 (1.6, 2.0)	1.8 (1.1, 2.2)	0.512
PSV (cm/s)	91.8 (58.4, 113.0)	114.0 (63.2, 133.0)	0.374
RI	0.71 (0.59, 0.76)	0.84 (0.80, 0.89)	**0**.008*
FV (ml/min)	41.8 (18.0, 60.1)	25.1 (9.9, 52.7)	0.314
T3			
Diameter (mm)	2.0 (1.8, 2.3)	1.9 (1.5, 2.2)	**0**.021*
PSV (cm/s)	76.4 (51.3, 86.7)	79.6 (64.7, 103.3)	0.139
RI	0.71 (0.65, 0.74)	0.84 (0.80, 0.88)	**0**.005*
FV (ml/min)	37.1 (20.0, 63.4)	21.9 (10.0, 44.4)	0.26

PSV, peak systolic velocity; RI, resistance index; FV, arterial blood flow volume; T1, pre-operation; T2, one week after operation; T3, three months after operation. *Represents *p* < 0.05, values of statistical significance are highlighted in bold.

From T2 to T3, CDUS showed that all bypass arteries remained patent. The diameter of the STA at the operative side continued to increase, and resistance remained low, while there were no significant changes in the diameter and RI of STA at the contralateral side. At T3, FV on both sides had slightly decreased compared to T2 (*p* > 0.05) but remained elevated compared to T1 (ipsilateral side *p* = 0.013, contralateral side *p* = 0.017). The STA on the operative side had an average diameter increase of 0.7 mm (54%) at T3 comparing with T1, with FV increased by approximately 26.0 ml/min (226%). The contralateral side had an average diameter increase of 0.4 mm (31%) at T3 compared to T1, with FV increased by about 15.7 ml/min (138%). At T3, there were statistically significant differences in both diameter and RI between the graft and contralateral sides, with the operative side had a larger diameter and significantly lower RI compared to the contralateral side (Figure 1 and Table 4).

#### Carotid artery (increased FV and PSV in ECA on the operation side with a time-dependent pattern)

**Figure 2:**
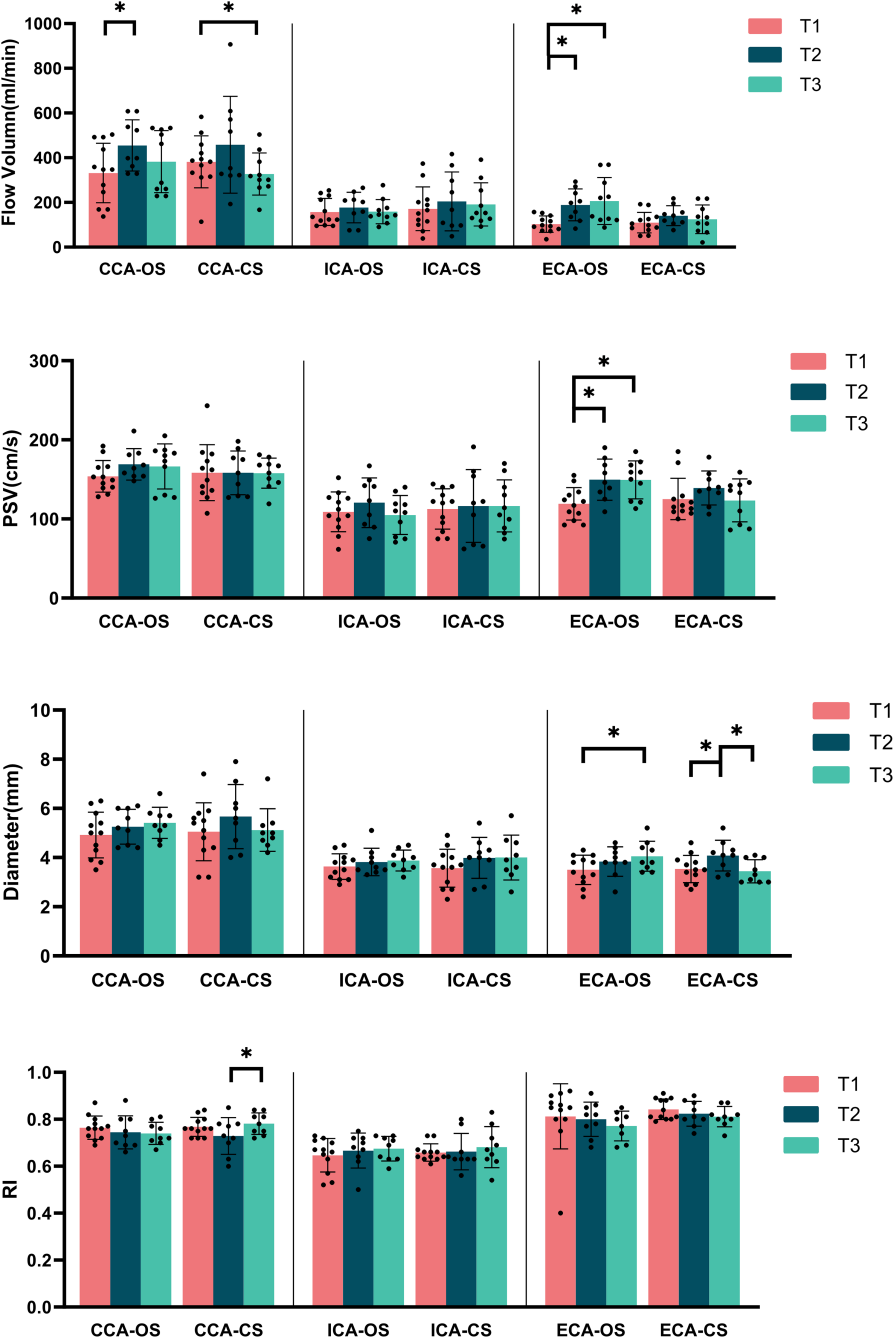
Carotid artery blood flow parameters at various time points in the MMD group (OS, operation side; CS, contralateral side; CCA, common carotid artery; ICA, internal carotid artery; ECA, external carotid artery; PSV, peak systolic velocity; RI, resistance index). *Represents *p* < 0.05.

From T1 to T3, there was a gradual increase in FV and PSV in the ECA at the operation side, with significant differences observed between T1 and T2 (FV: *p* = 0.028, PSV: *p* = 0.011) as well as between T1 and T3 (FV: *p* = 0.005, PSV: *p* = 0.007). Additionally, the diameter of the ECA on the operation side also showed an increase over time, with a significant difference noted between T1 and T3 (*p* = 0.033). In contrast, on the contralateral side, the ECA diameter increased at T2 but then decreased at T3, nearly returned to the levels observed at T1. This change was statistically significant between T1 and T2 (*p* = 0.012) and between T2 and T3 (*p* = 0.027). However, no significant differences in RI were found for the ECA at any of the three time points (Figure 2).

At T2, the FV in the CCA on the operation side was significantly higher compared to T1 (*p* = 0.015), although it slightly decreased at T3 while remained above the levels observed at T1. The contralateral side showed a similar trend; however, the FV at T3 was lower than that at T1, with a statistically significant difference between T1 and T3 (*p* = 0.009). Notably, there were no significant differences in the PSV and diameter for the CCA across any of the three time points on either side (Figure 2). No significant differences were found in artery diameter, FV, PSV or RI of bilateral ICA at any of the three time points (Figure 2).

### STA blood flow increase percentage correlation with matsushima grading (the increase in STA blood flow is negatively correlated with matsushima grading)

**Figure 3:**
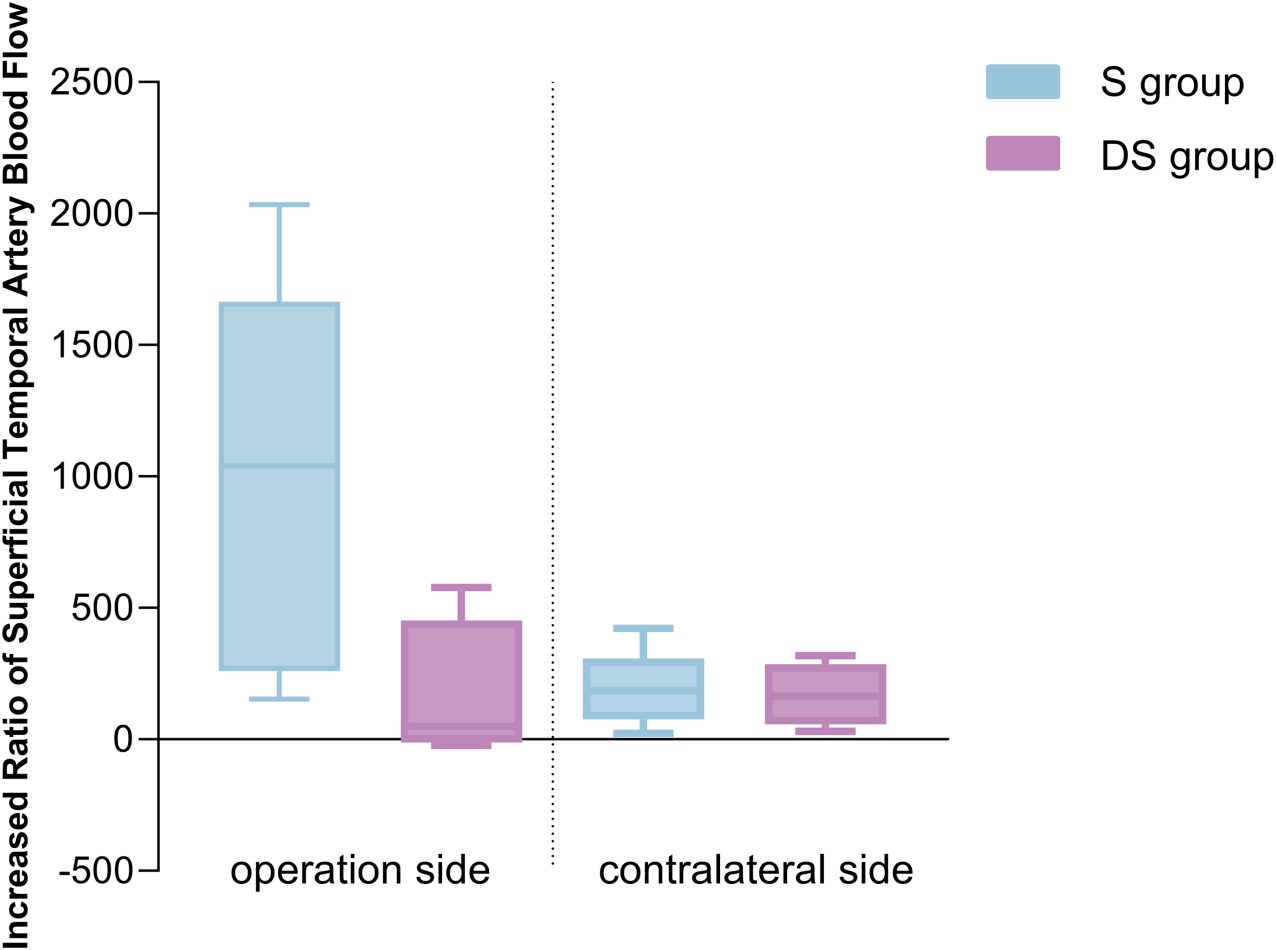
STA blood flow increasing percentage correlation with matsushima grading.

**Figure 4:**
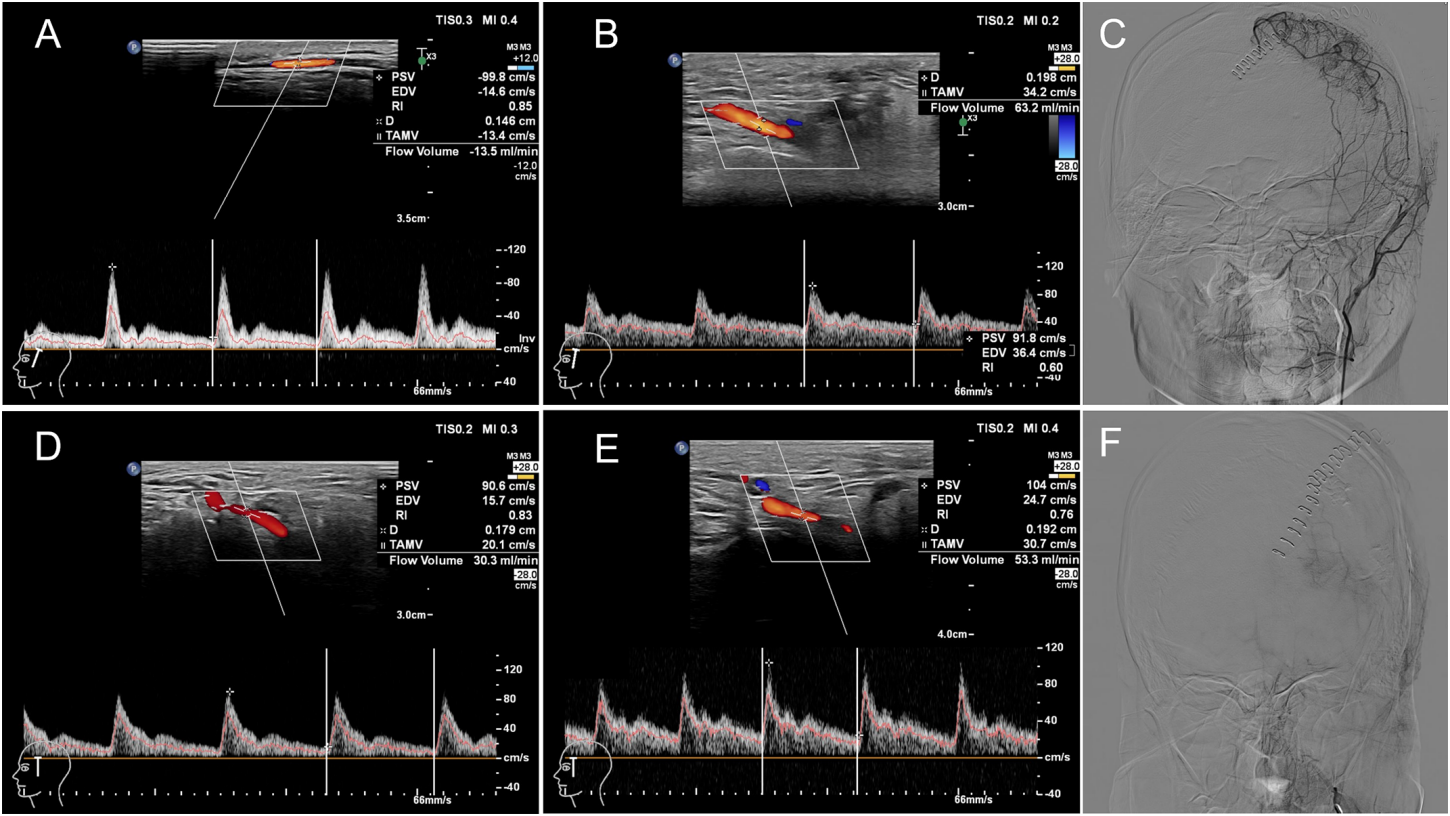
CDUS and DSA images of two children with MMD undergoing revascularization surgery on their left hemispheres. Patient 1: A 16-year-old male patient in S group (satisfactory compensation). The flow volume of the left STA was 13.5 ml/min **(A)** and 63.2 ml/min **(B)** preoperatively and at 7 days postoperatively respectively with the percentage increased in blood flow of STA was 368.15%. The Matsushima grade was B by DSA **(C)**. Patient 2: A 15-year-old female patient in DS group (dissatisfactory compensation). The flow volume of the left STA was 30.3 ml/min **(D)** and 53.3 ml/min **(E)** preoperatively and at 7 days postoperatively respectively with the percentage increased in blood flow of STA was 75.91%. The Matsushima grade was C by DSA **(F)**. PSV: peak systolic velocity, EDV: end diastolic velocity, RI: resistance index, D: artery diameter, TAMV: time averaged mean flow velocity.

Based on postoperative DSA angiography, all Moyamoya patients at T2 were graded according to the Matsushima grading, which categorized them into two groups: 6 cases in the S group and 6 in the DS group. The percentage increase in STA blood flow on the operative side in the S group at T2 was higher than that in the DS group (Figure 3), with a correlation between the percentage increase in blood flow and Matsushima grading (Spearman rho: −0.693, *p* = 0.039). This suggested that a greater increase in STA FV at T2 corresponds to a lower Matsushima grade, indicating a wider distribution of collateral circulation (Figure 4). However, there were no statistically significant differences in the percentage increase in FV between the operation and contralateral STA in both groups.

Patient 1: A 16-year-old male patient in S group. The FV of the left STA was 13.5 ml/min (A) and 63.2 ml/min (B) preoperatively and at 7 days postoperatively respectively with the percentage increased in blood flow of STA was 368.15%. The Matsushima grade was B by DSA(C). Patient 2: A 15-year-old female patient in DS group. The FV of the left STA was 30.3 ml/min (D) and 53.3 ml/min (E) preoperatively and at 7 days postoperatively respectively with the percentage increased in blood flow of STA was 75.91%. The Matsushima grade was C by DSA (F).

## Discussion

MMD is characterized by the progressive narrowing and blockage of intracranial vessels, particularly from the terminal internal carotid artery to the middle and anterior cerebral arteries. This condition leads to a shift in the brain's blood supply, moving away from the ICA system and relying more on the ECA and vertebral artery systems for compensation. Studies by Shuai Zheng et al. (10) have shown that in adult patients with MMD, there is a progressive decline in the diameter, PSV, end-diastolic velocity, and FV of the ICA as Suzuki stages advance. This study revealed a comparable pattern in children with MMD. The comparison of FV using CDUS between the MMD and control hemispheres directly indicated a diminished blood supply to the anterior circulation in children with MMD, with a particularly notable reduction in the ICA supply. The observed increase in PSV in the CCA, ECA, and STA on the affected side may suggest a compensatory response of the ECA system to maintain adequate blood supply.

Surgical revascularization techniques frequently employed in clinical practice encompass direct revascularization, indirect revascularization, and hybrid approaches that integrate both methods (11, 12). Direct revascularization procedures, exemplified by the STA-MCA bypass surgery, facilitate an immediate augmentation of local cerebral blood flow via the bypass artery. In contrast, indirect revascularization techniques, such as encephalo-duro-arterio-synangiosis, encephalo-myosynangiosis, multiple burr hole surgery, and dural inversion, promote angiogenesis by fostering the development of new vascular connections between the cerebral surface and donor tissues with established vascularization. Recent studies on children with MMD have demonstrated that long-term outcomes following revascularization surgery are generally favorable, but further investigation is needed to determine the most appropriate surgical techniques and indications (1, 2). CDUS is a crucial imaging assessment method for pediatric patients. This study illustrated that in children with MMD undergoing combined revascularization surgery, CDUS conducted preoperatively, at 7 days postoperatively, and at 3 months postoperatively, yielded extensive data regarding the hemodynamic alterations in the carotid and superficial temporal arteries. As a bypass artery, the modifications observed in the STA were indicative of the transition from extracranial to intracranial blood supply. Initially functioning as a medium-resistance artery providing blood to the superficial tissues of the head, the STA on the operation side underwent an immediate transformation into a low-resistance artery supplying the intracranial arteries following combined revascularization surgery. The RI of the STA significantly decreased in comparison to both the contralateral side and its preoperative state. This reduction persisted at three months postoperatively, serving as direct evidence of successful direct revascularization. These findings were consistent with outcomes observed in MMD patients undergoing revascularization (7, 13). Furthermore, the increased diameter, enhanced PSV and a swift elevation in FV of STA at T2 further substantiate the efficacy of direct revascularization, paralleling outcomes observed in MMD population (6, 13, 14). As a result, some researchers suggested that quantitative ultrasound evaluations may serve as a feasible alternative to conventional DSA follow-ups following STA bypass surgery (8).

From 7 days to 3 months following surgery, both the PSV and FV of the STA exhibited a decline to varying extents; however, the FV remained elevated compared to preoperative values. Notably, this FV level was significantly lower than that observed in adults three months post-combined revascularization surgery and was also lower than in patients who underwent solely direct revascularization (8, 14). Chen S et al. indicate that an increase in STA blood flow exceeding 69.5 ml/min three months post-surgery may serve as a predictor of inadequate neovascularization at the six-month mark (7). In contrast to direct revascularization surgery, which solely relies on the immediate blood supply provided by the bypass graft, combined revascularization surgery encompasses both this direct supply and the subsequent gradual development of new blood vessels on the brain surface postoperatively. Nonetheless, the brain's total blood supply capacity is limited, the benefits of indirect revascularization may counteract those achieved through direct revascularization. Consequently, the observed decrease in STA blood flow postoperatively may be partially attributable to the progressive increase in neovascularization. Nonetheless, the precise timeline for this phenomenon varies across different studies (7–9, 14), potentially due to variations in surgical techniques and individual patient characteristics. The majority of existing literature predominantly addresses adult or mixed populations, with a notable paucity of studies specifically examining children with MMD. Therefore, a comprehensive evaluation of hemodynamic compensation in children undergoing combined revascularization surgery requires more than just assessing the bypass artery.

The STA is a branch of the ECA, and hemodynamic changes in the STA inevitably lead to corresponding changes in the ECA. Additionally, the collateral compensation achieved through indirect revascularization after combined revascularization surgery is partly realized by compensation from other branches of the ECA, such as the middle meningeal artery and deep temporal artery (5, 7, 15). Studies have shown that the PI ratio of ECA/ICA and PI of ECA at three months postoperatively can serve as one of the indicators predicting well-developed neovascularization and better prognosis in adults with ischemic MMD who undergo combined revascularization surgery (5). This study lacks long-term observational indicators, but in children with successful revascularization at T3, increased diameter, higher PSV, and increased FV of ECA compared to T1 was showing at the surgical side, which may be evidence of the indirect revascularization starting to take effect. The ECA could be an important indicator for further research in the assessment of surgical outcomes after combined revascularization surgery in the future.

Complications following combined revascularization surgery mainly include transient ischemic attacks, ischemic strokes (both ipsilateral and contralateral), hyperperfusion syndrome, cerebral hemorrhage, and epilepsy (16–19). Many researchers (20–22) suggested that postoperative hemodynamic fluctuations, such as the “watershed shift” phenomenon, were key contributors to these complications. In our study, we observed a decrease in FV of the ICA on the surgical side or in the contralateral in some children, or a sharp increase in FV of the bypass artery at T2. These changes may serve as potential risk factors for ischemic stroke or hyperperfusion. Quantitative measurement of ultrasound hemodynamics may help clinicians better understand these changes before and after combined revascularization surgery in children, allowing for more precise blood pressure control. Nonetheless, further research with larger sample sizes is essential to validate these findings.

This study had a small sample size due to the rarity MMD and the inability of some pediatric patients to undergo surgery or complete all ultrasound and DSA examinations. Furthermore, the follow-up period was relatively brief, which may not have allowed sufficient time for the full effects of indirect revascularization to manifest. Future research should include longer-term ultrasound follow-ups in a larger cohort of children with MMD.

## Conclusion

Preoperative and postoperative evaluation of hemodynamics in the carotid artery and STA using CDUS indicated an enhanced blood flow from ECA system to intracranial circulation following revascularization surgery. This enhancement was demonstrated by increases in arterial diameter, PSA and FV, along with a reduction in the RI of STA. Notably, increased FV in the STA at T2 was associated with Matsushima grading, suggested that CDUS might be a valuable tool for evaluating intracranial hemodynamic changes in children with MMD who underwent revascularization. Furthermore, CDUS holds promise for predicting the compensatory effects of collateral circulation, offering a noninvasive complement to traditional imaging methods. Its significant potential for clinical application in children with MMD warrants further investigation to establish its role in standard care practices.

## Data Availability

The raw data supporting the conclusions of this article will be made available by the authors, without undue reservation.

## References

[1] Morshed RA Abla AA Murph D Dao JM Winkler EA Burkhardt J-K Clinical outcomes after revascularization for pediatric moyamoya disease and syndrome: a single-center series. J Clin Neurosci. (2020) 79:137–43. 10.1016/j.jocn.2020.07.016 33070883 PMC7573194

[2] Gonzalez NR Amin-Hanjani S Bang OY Coffey C Du R Fierstra J Adult moyamoya disease and syndrome: current perspectives and future directions: a scientific statement from the American heart association/American stroke association. Stroke. (2023) 54(10):e465–79. 10.1161/STR.0000000000000443 37609846

[3] Bersano A Khan N Fuentes B Acerbi F Canavero I Tournier-Lasserve E European stroke organisation (ESO) guidelines on moyamoya angiopathy endorsed by vascular European reference network (VASCERN). Eur Stroke J. (2023) 8(1):55–84. 10.1177/23969873221144089 37021176 PMC10069176

[4] Nishizawa T Fujimura M Katsuki M Mugikura S Tashiro R Sato K Prediction of cerebral hyperperfusion after superficial temporal artery-middle cerebral artery anastomosis by three-dimensional-time-of-flight magnetic resonance angiography in adult patients with moyamoya disease. Cerebrovasc Dis. (2020) 49(4):396–403. 10.1159/000509740 32829323

[5] Matsuo S Amano T Miyamatsu Y Yamashita S Yasaka M Okada Y Carotid ultrasonography predicts collateral development following combined direct and indirect revascularization surgery in adult ischemic moyamoya disease. Clin Neurol Neurosurg. (2021) 203:106590. 10.1016/j.clineuro.2021.106590 33711640

[6] Connolly F Alsolivany J Czabanka M Vajkoczy P Valdueza JM Röhl JE Blood volume flow in the superficial temporal artery assessed by duplex sonography: predicting extracranial-intracranial bypass patency in moyamoya disease. J Neurosurg. (2021) 135(6):1666–73. 10.3171/2020.9.JNS202709 33836503

[7] Chen S Wang B Wen Y Wang Z Long T Chen J Ultrasonic hemodynamic changes of superficial temporal artery graft in different angiogenesis outcomes of moyamoya disease patients treated with combined revascularization surgery. Front Neurol. (2023) 14:1115343. 10.3389/fneur.2023.1115343 36873438 PMC9978192

[8] Wang G Zhang X Wang B Wen Y Chen S Liu J Flow evaluation of STA-MCA bypass using quantitative ultrasonography: an alternative to standard angiography for follow up of bypass graft. J Stroke Cerebrovasc Dis. (2020) 29(9):105000. 10.1016/j.jstrokecerebrovasdis.2020.105000 32807419

[9] Takahashi S Toda M. Usefulness of STA ultrasonography parameters after STA-MCA bypass in patients with moyamoya disease: a short review. Neurosurg Rev. (2024) 47(1):26. 10.1007/s10143-023-02262-3 38163827

[10] Zheng S Ge P Shi Z Wang J Li Y Yu T Clinical significance of ultrasound-based hemodynamic assessment of extracranial internal carotid artery and posterior cerebral artery in symptomatic and angiographic evolution of moyamoya disease: a preliminary study. Front Neurol. (2021) 12:614749. 10.3389/fneur.2021.614749 34079508 PMC8165238

[11] Li Y Wang AR Steinberg GK. Abstract MP47: clinicoradiological outcomes of surgical revascularization for moyamoya disease with minimum 10-year follow-up. Stroke. (2021) 52(1):AMP:47. 10.1161/str.52.suppl_1.MP47

[12] Onodera K Ooigawa H Tabata S Kimura T Lepic M Suzuki K Effect of revascularization surgery on cerebral hemodynamics in adult moyamoya disease. Clin Neurol Neurosurg. (2024) 239:108180. 10.1016/j.clineuro.2024.108180 38452713

[13] Wang JZ Mu J Zhang D Zheng S Zhu X Wei X. Clinical use of color Doppler ultrasonography to predict and evaluate the collateral development of two common revascularizations in patients with moyamoya disease. Front Neurol. (2022) 13:976695. 10.3389/fneur.2022.976695 36388226 PMC9649901

[14] Wen Y Gou Y Wang B Wang Z Chen S Zhang S Is STA really a low-flow graft? A quantitative ultrasonographic study of the flow of STA for cerebral revascularization in MMD patients. CNS Neurosci Ther. (2023) 29(9):2572–82. 10.1111/cns.14197 37002791 PMC10401118

[15] Shen W Xu B Li H Gao X Liao Y Shi W Enlarged encephalo-duro-myo-synangiosis treatment for moyamoya disease in young children. World Neurosurg. (2017) 106:9–16. 10.1016/j.wneu.2017.06.088 28645592

[16] Sato D Miyawaki S Imai H Hongo H Kiyofuji S Koizumi S Clinical characteristics of immediate contralateral ischemia subsequent to revascularization for moyamoya disease. World Neurosurg. (2024) 183:e355–65. 10.1016/j.wneu.2023.12.100 38154683

[17] Sussman ES Madhugiri V Teo M Nielsen TH Furtado SV Pendharkar AV Contralateral acute vascular occlusion following revascularization surgery for moyamoya disease. J Neurosurg. (2019) 131(6):1702–8. 10.3171/2018.8.JNS18951 30554188

[18] Kim HG Lee SK Lee JD. Characteristics of infarction after encephaloduroarteriosynangiosis in young patients with moyamoya disease. J Neurosurg Pediatr. (2017) 19(1):1–7. 10.3171/2016.7.PEDS16218 27715484

[19] Okuyama T Kawabori M Ito M Sugiyama T Kazumata K Fujimura M. Outcomes of combined revascularization surgery for moyamoya disease without preoperative cerebral angiography. World Neurosurg. (2022) 165:e446–51. 10.1016/j.wneu.2022.06.067 35750140

[20] Tu X-k Fujimura M Rashad S Mugikura S Sakata H Niizuma K Uneven cerebral hemodynamic change as a cause of neurological deterioration in the acute stage after direct revascularization for moyamoya disease: cerebral hyperperfusion and remote ischemia caused by the ‘watershed shift’. Neurosurg Rev. (2017) 40(3):507–12. 10.1007/s10143-017-0845-9 28357585

[21] Hara S Nariai T Inaji M Tanaka Y Maehara T. Imaging pattern and the mechanisms of postoperative infarction after indirect revascularization in patients with moyamoya disease. World Neurosurg. (2021) 155:e510–21. 10.1016/j.wneu.2021.08.098 34464770

[22] Wang J Jiang H Tang J Lin C Ni W Gu Y. Postoperative cerebral infarction after revascularization in patients with moyamoya disease: incidence and risk factors. Front Neurol. (2022) 13:1053193. 10.3389/fneur.2022.1053193 36479051 PMC9720261

